# Fluorene intercalated graphene oxide based CoQ10 imprinted polymer composite as a selective platform for electrochemical sensing of CoQ10†

**DOI:** 10.1039/d2ra05401a

**Published:** 2022-11-04

**Authors:** Anam Naz Soomro, Huma Shaikh, Muhammad Imran Malik, Jamil A. Buledi, Sehrish Qazi, Amber Solangi

**Affiliations:** National Center of Excellence in Analytical Chemistry, University of Sindh Jamshoro-76080 Sindh Pakistan huma.hashu@gmail.com soomroanamnaz@gmail.com +92-022-2771560 +92-022-2771379; H. E. J. Research Institute of Chemistry, International Centre for Chemical and Biological Sciences (ICCBS), University of Karachi Karachi-75270 Sindh Pakistan mimran.malik@iccs.edu

## Abstract

The new objective of sustainable analytical chemistry is to develop validated robust, swift, simple and highly sensitive analytical methods that are based on cost effective sensing technology. Therefore, in this study the electro-chemical detection of coenzyme Q10 (CoQ10) was achieved using a fluorene intercalated graphene oxide based CoQ10 imprinted polymer composite modified glassy carbon electrode (CoQ10-IGOPC/GCE). The synthesized sensing material was characterized using SEM, XRD and FT-IR to determine the morphology and functional properties. The CoQ10-IGOPC/GCE was characterized by EIS for its electrochemical properties. CoQ10 was detected selectively using Differential Pulse Voltammetry (DPV). Under ideal circumstances, a linear calibration curve with a correlation coefficient (*R*^2^) of 0.991 was produced in the concentration range of 0.0967 to 28.7 μM. The limit of detection (LOD) and limit of quantification (LOQ) were found to be 0.029 and 0.0967 μM, respectively. Furthermore, the proposed electrochemical sensor was extremely selective, accurate and thoroughly validated with RSD values less than 5%. The developed CoQ10-IGOPC/GCE based electrochemical sensor was successfully used for the detection of CoQ10 in samples of fruits, vegetables, nuts, human blood serum and pharmaceuticals. The CoQ10-IGOPC/GCE based electrochemical method showed good percent recoveries ranging from 94 to 103 percent.

## Abstract 2

The new objective of sustainable analytical chemistry is to develop validated robust, swift, simple and highly sensitive analytical methods that are based on cost effective sensing technology.

## Graphical abstract

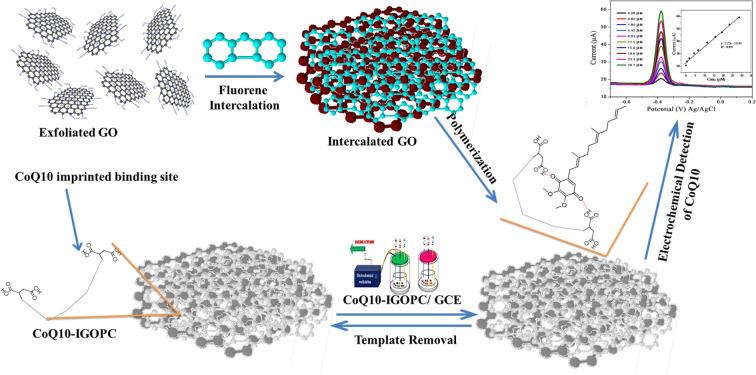

## 1. Introduction

Ubiquinone (coenzyme Q) is an endogenous hydroxyl benzoquinone molecule that is fat soluble and found mostly in aerobic organisms.^1^ It is found in two different redox states that possess isoprenoid units of variable chain length. These isoprenoid units are involved in electron transfer as electron carriers during the energy production process within mitochondrial oxidative phosphorylation.^2^ The most common type of coenzyme Q is coenzyme Q10 (CoQ10) which is found in humans and animals. It has 10 isoprenoid units on the side chain.^3^ Human serum and blood tissues contain 90% of the CoQ10 content in the reduced state called ubiquinol-10 (CoQ10H_2_). CoQ10H_2_ is very sensitive and liable to oxidation. However, it is a robust fat-soluble antioxidant that safeguards DNA.^4^ On the other hand the oxidized form (ubiquinone) may enzymatically be reduced to ubiquinol after absorption.^5^ It secures phospholipids of membranes from peroxidation and the phenomenon has been applied in many tissue injury mechanisms.^6^ Coenzyme Q10 is a successful scavenger of free radicals due to which it is able to detoxify cell toxicity by participating in many elimination mechanisms of waste products that are produced during the metabolic reactions.^7^ The heart, liver, kidneys, muscles, pancreas and spleen contain large amounts of CoQ10.^8^ CoQ10 has been recommended by the nutritionists as supplement during cancer and cardiovascular diseases treatment because human body decreases its production with aging. Thus, there is need to monitor its presence in human body, dietary supplements, fruits, vegetables *etc. via* extraordinary selective, cost effective, robust and swift analytical methods. Most of the determination techniques such as HPLC,^9^ LC-MS,^10^ FTIR,^11^*etc.*, are tedious, time-consuming, expensive, require pre-concentration of sample and utilize multi-solvent extraction procedures before analysis. Thus, there is a need to fill this gap by developing simple, sensitive, swift, cost effective and accurate analysis methods for CoQ10 that should overcome the disadvantage of conventional techniques such as HPLC, HPLC-MS, *etc.* The antioxidant property of CoQ10 in various reactions of body has made its place to study and investigate its electrochemical properties. As CoQ10 was found electrochemically active; it was feasible to determine CoQ10 from different matrices using facile, swift and cost effective electrochemical methods.^12,13^ Depending on the electrolyte used, electrochemical sensors can be used in real matrices at range of temperatures.^14^ When compared to other analytical techniques, the voltammetry methods stand out for their simplicity, speed, and sensitivity, as well as their exceptional potential for urgency and rapid response detection.^15^ The primary goal of our research was to develop a robust electrochemical method using voltammetry for sensitive detection of CoQ10 in real samples of diverse nature.

Molecular imprinting is a type of biomimetics that involves using a molecular template to influence the development of a synthetic polymer with particular recognition sites.^16^ Among other applications, MIPs have been used in sensors, chromatography, immunoassays, controlled medication delivery, and catalysis.^17,18^ Specificity is heart of sensor technology and incorporating MIPs with sensing materials or preparing MIP based sensing materials gives boost to the sensitivity and specificity of sensor. Therefore, they are producing promising results when used as electrochemical sensing materials.^19,20^

Graphene is a promising platform for the electrochemical detection of analytes.^21^ Graphene and its derivatives perform swift electrochemical reactions due to their high surface area, fast electron transfer kinetics and excellent chemical, mechanical and thermal properties.^22^ Graphene and reduced graphene oxide due to their excellent conductivity and response to electrochemical stimuli lead to highly sensitive electrochemical sensors.^23^ Therefore, the formation of molecular imprinted polymer on the sheets of graphene derivatives leads to the composite material that is highly conductive due to 2D sheets of graphene and highly selective due to the presence of specific binding sites in the MIPs. This hyphenation results in production of highly sensitive and selective electrode material that can undergo fast electrochemical reaction along with good selectivity in complex real matrices.

In the present study, we explored the electrochemical analysis of CoQ10 using CoQ10-IGOPC/GCE *via* Differential Pulse Voltammetry (DPV). Electrochemical impedance spectroscopy verified that CoQ10-IGOPC/GCE has outstanding electrochemical characteristics. The method was thoroughly validated, and tested for CoQ10 samples of diverse matrices. The results revealed that developed CoQ10-IGOPC based electrochemical method is highly robust and selective. It can be utilized for the determination of CoQ10 in biological, fruits, vegetables and CoQ10 supplement samples.

## 2. Experimental section

### 2.1. Chemicals and reagents

All chemicals used were of analytical grade. Graphite flakes, acetic acid (99.7%), sulphuric acid (97.0%), sodium hydroxide (98%), acetone (99.5%), KMnO_4_ (99.0%), NaNO_3_ (99.0%), H_2_O_2_ (30%), and methanol (99.9%) were purchased from Sigma Aldrich USA. Itaconic acid (99%), azobisisobutyronitrile (98%), standard of coenzyme Q10 (98%), fluorene (98%), acetonitrile (99.8%), *n*-hexane (97.0%), 2-propanol (%), ethanol (96%), NaCl (99.5%), ascorbic acid (99.0%), tetrahydrofuran (THF) (99.9%), and phosphoric acid (85%) were bought from MERCK Germany. All of the experimental solutions were made with deionized (DI) water.

### 2.2. Instrumentation and apparatus

X-ray diffractometer (XRD) model XRD-7000-Shimadzu was used to investigate the crystalline characteristics of produced CoQ10-IGOPC. A scanning electron microscope (SEM) SEM-JSM 7800F was used to analyze the surface morphology of synthesized material. Fourier transform infrared (FT-IR) spectroscopy (Nicolet-5700, Thermo Finnigan, USA) and UV. Visible spectroscopy (Lambda-35, PerkinElmer, USA) were used to identify the different functionalities of the synthesized material. Electrochemical work station CH-was used for method development.

### 2.3. Synthesis of CoQ10 imprinted graphene oxide based polymeric composite (CoQ10-IGOPC) and non-imprinted graphene oxide based polymeric composite (NIGOPC)

A modified Hummers' method was used to prepare graphene oxide (GO).^24^ The prepared GO was subjected to intercalation using fluorene. Precisely, GO suspension was prepared by taking 100 mg of GO in 10 ml of DI water and sonicated for 30 minutes at ambient temperature. Then the intercalation of fluorene into the layers of GO was carried by mixing 0.072 M (300 mg in 25 ml of acetone) fluorene solution in prepared GO suspension. The mixture was sonicated for 4 hours for proper intercalation and washed twice with acetone to remove unreacted fluorene, and finally dried at room temperature. Then 100 mg of dried fluorene intercalated GO (Fl Int. GO) was dispersed in 5 ml of acetonitrile and mixed with 1.15 × 10^−3^ M (5 mg in 5 ml acetonitrile) solution of CoQ10. Both solutions were mixed and stirred for 2 hours to allow the pre-polymerization complexation between CoQ10 and Fl Int. GO. After 2 hours 0.19 M (130 mg in 5 ml acetonitrile) of itaconic acid and 6.09 × 10^−3^ M (1 mg in 1 ml acetonitrile) of azobisisobutyronitrile were added (as initiator) and mixture was heated at 60 °C for 24 hours with continuous stirring. The successfully synthesized CoQ10 imprinted graphene oxide based polymeric composite (CoQ10-IGOPC) was washed with mixture of acetic acid and methanol (1 : 3) until the template was completely removed. However the non-imprinted graphene oxide based polymeric composite (NIGOPC) was synthesized following same procedure without addition of CoQ10.

### 2.4. Modification of glassy carbon electrode (GCE)

The CoQ10-IGOPC was immobilized on the electrode's surface using the drop casting process. To make the drop casting suspension, 10 mg of CoQ10-IGOPC was suspended in 2 ml DI water and 100 μl of 5% Nafion solution was used as a binder, later for 20 minutes it was sonicated. The thoroughly cleaned and glossed surface of the GCE was modified using 10 μl of prepared suspension by drop and cast method. Finally, the successfully fabricated CoQ10-IGOPC/GCE was used to conduct electrochemical tests for selective determination of CoQ10. Same procedure was used to prepare the NIGOPC/GCE.

### 2.5. Electrochemical procedure

Electrochemical measurements were performed using an electrochemical analyzer (CHI 760 Electrochemical Workstation USA equipped with CHI 760 software) with three electrode system in 10 ml electrochemical cell. The electrode system involved a working electrode (CoQ10-IGOPC/GCE), whose current was the function of analytes concentration, a counter electrode (platinum wire) for passage of current, and a reference electrode (Ag/AgCl, 2 M) which served as a standard. The CoQ10-IGOPC/GCE, NIGOPC/GCE and bare GCE were characterized by electrochemical impedance spectrometry (EIS). The impedance investigation was carried out at room temperature (20 °C) in the frequency range of 100 kHz to 100 MHz. All electrochemical quantifications were recorded at optimized conditions: mode; DPV (differential pulse voltammetry), electrolytic solution; NaOH of pH 12, amplitude; 0.05 V, quiet time; 2.0 seconds, pulse width; 0.05 seconds, sample width; 0.0167 seconds, pulse period; 0.2 seconds. Before each analysis the electrodes were washed with DI water. The real samples were extracted as explained in ESI Section 2.1.†

## 3. Results and discussion

### 3.1. Synthesis of CoQ10-IGOPC

The strategy to produce nanolayer of MIP into the layers of GO includes the intercalation of GO with fluorene. Fluorene and its derivative have been explored extensively for their applications in the fabrication of blue polymer, OLED, OSC, *etc.*^25^ There are several reports about the homo- and co-polymers of fluorene that show excellent conductivity and electrochromic properties.^26^ Therefore, fluorene was intercalated into the sheets of GO as functional monomer to facilitate the pre-polymerization complexation between Fl Int. GO and CoQ10. It is because the effective complexation between functional monomer and template leads to the highly specific MIP. The itaconic acid was introduced as crosslinking monomer to produce CoQ10 imprinted poly (fluorene-*co*-itaconic acid) into the layers of GO. The resulting polymeric composite was highly conductive and specific for CoQ10.

### 3.2. Characterization of synthesized material

**Fig. 1:**
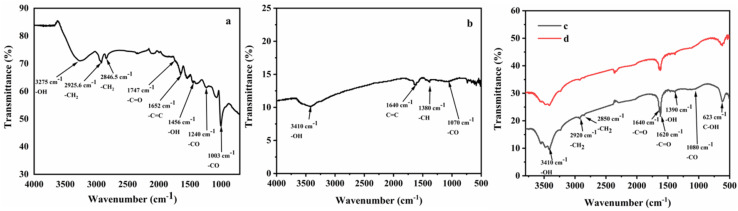
FTIR spectrum of (a) GO, (b) Fl Int. GO, and (c) CoQ10-IGOPC and (d) NIGOPC.

FTIR was used to identify the functionalities of the materials. In Fig. 1a the FTIR spectrum of GO shows several peaks indicating oxygen containing functionalities which justify that graphite was successfully oxidized. A broad peak was observed at 3275 cm^−1^ due to –OH stretching vibrations. The peaks at 2925 cm^−1^ and 2846.5 cm^−1^ were observed due to –CH_2_ stretching vibrations of graphene. The stretching vibration of the C=O group was observed at 1747 cm^−1^ and aromatic stretching peak of C=C group appeared around 1652 cm^−1^. The peaks at 1240 cm^−1^ and 1003 cm^−1^ were due to C–O stretching. All these peaks were GO's characteristic peaks. In the Fig. 1b, a broad peak appeared at 3410 cm^−1^ due to –OH stretching vibrations. The peak at 1640 cm^−1^ represents C=C stretching vibrations. Furthermore, a weak peak at 1070 cm^−1^ was observed due to –CO stretching. A new peak appeared at 1380 cm^−1^ due to C–H bending that indicated the successful interaction of fluorene within the sheets of GO. The weak intensities of peaks representing oxygen containing functionalities in Fl Int. GO suggested that after intercalation process the oxygen containing functionalities of GO were less dominant. Moreover, fluorene interacted with graphene sheets *via* π–π interactions. Fig. 1c and d represent FTIR spectra of CoQ10-IGOPC and NIGOPC, respectively. Both the spectra are similar due to same material backbone. The broad peak with multiple steps at 3410 cm^−1^ is the characteristic peak of itaconic acid due –OH stretching vibrations. The –CH_2_ stretching vibrations were observed at 2920 cm^−1^ and 2850 cm^−1^. The peaks at 1640 cm^−1^ and 1620 cm^−1^ were also characteristic peaks of itaconic acid due to the presence of carbonyl functionalities. A new peak appeared at 623 cm^−1^ due to C–OH functionality in itaconic acid. The FTIR spectra of CoQ10-IGOPC and NIGOPC clearly indicated that surface of Fl Int. GO was successfully modified with CoQ10 imprinted poly (fluorene-*co*-itaconic acid) and non-imprinted poly (fluorene-*co*-itaconic acid).

**Fig. 2:**
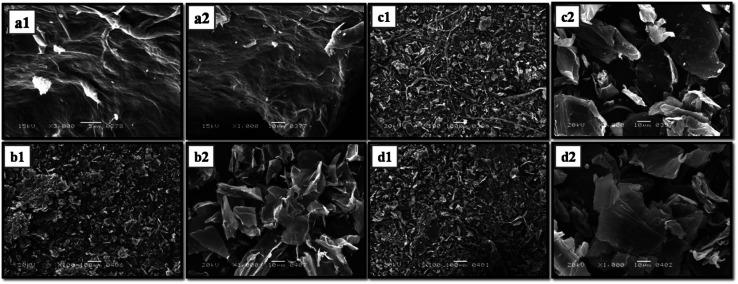
SEM image of (a1 and a2) GO, (b1 and b2) Fl Int. GO, (c1 and c2) CoQ10-IGOPC and (d1 and d2) NIGOPC.

A scanning electron microscope examination was carried out to find out the surface morphology of CoQ10-IGOPC. In Fig. 2a1 and a2 it has been demonstrated that GO has a smooth, layered, and two-dimensional sheet-like structure. The films were layered one on top of the other and had wrinkles. Because the oxygen-containing functional groups were predominantly coupled at the margins of GO sheets, the sheets were found to be thick at the edges.^27^ With the addition of fluorene the roughness of the Fl Int. GO was increased as compared to the GO as given in Fig. 2b1 and b2, that justified the intercalation of fluorene into the layers of GO. Moreover, the surface of CoQ10-IGOPC had thread like structures (Fig. 2c1 and c2) which indicated the polymerization reaction and the small interconnected granules seemed to be composed of polymer particles.^28^ The Fig. 2d1 and d2 indicated the polymeric granular structure of NIGOPC with thread like appearance.

**Fig. 3:**
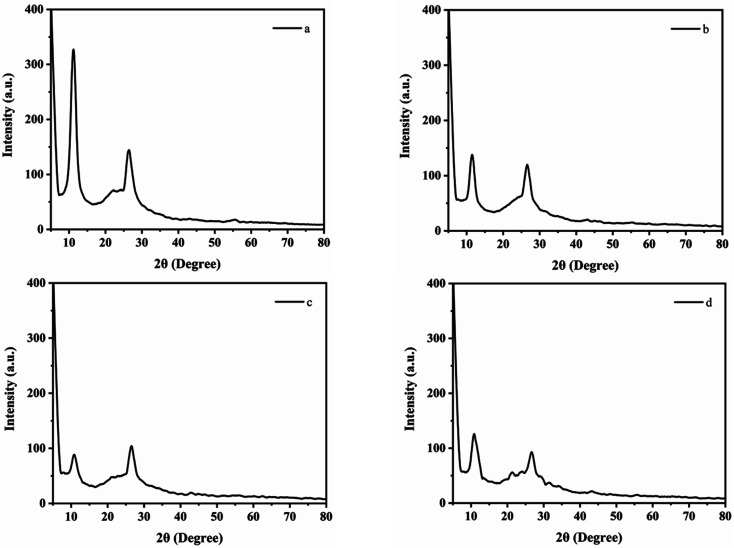
XRD spectrum of (a) GO, (b) Fl Int. GO, (c) CoQ10-IGOPC, and (d) NIGOPC.

X-ray diffraction spectroscopy is the characterization technique used to determine the atomic and molecular structure of crystal materials.^29^ The XRD spectrum of GO showed an intense diffraction peak at 2*θ* = 11.2° with an interlayer distance of 7.9 Å along with the appearance of small non-oxidized graphene peak at 2*θ* = 26.1° with *d* spacing of 3.5 Å (Fig. 3a). The interlayer gap had increased from 3.5 Å to 7.9 Å for graphene oxide due the introduction of different functional groups as a result of oxidation. The XRD spectrum of fluorene intercalated GO (Fl Int. GO) showed the diffraction peak at 2*θ* = 11.4° and *d* spacing of 7.8 Å (Fig. 3b). Moreover the intensity of the diffraction peak had been reduced that justified that the interaction of fluorene into GO sheets reduced the crystalline structure of GO. Fig. 3c explained the XRD spectrum of CoQ10-IGOPC with diffraction peak of 2*θ* = 10.8° with interlayer spacing of 8.1 Å. This increase in interlayer spacing indicated the presence of nanolayer of MIP within the layers of GO. While in Fig. 3d the diffraction peak at 2*θ* = 10.6° also confirmed the presence nanolayer of NIP within the layers of GO.

Debye–Scherer equation was used to compute the sheet height of the synthesized material.
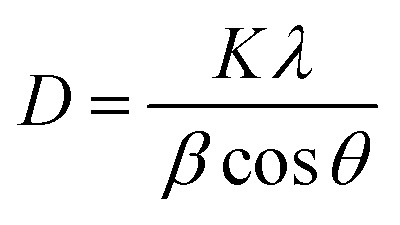
where *D* is the crystallite height in the direction perpendicular to the lattice planes, *K* is a numerical factor commonly referred to as the crystallite-shape factor 5, *λ* is the X-ray wavelength, *β* is the width (full-width at half-maximum) of the X-ray diffraction peak in radians and *θ* is the Bragg angle. The calculated sheet height of GO, Fl Int. GO, NIGOPC and CoQ10-IGOPC was obtained as 1.7, 0.8, 0.51 and 0.52 nm, respectively.

### 3.3. Electron impedance spectroscopy (EIS)

**Fig. 4:**
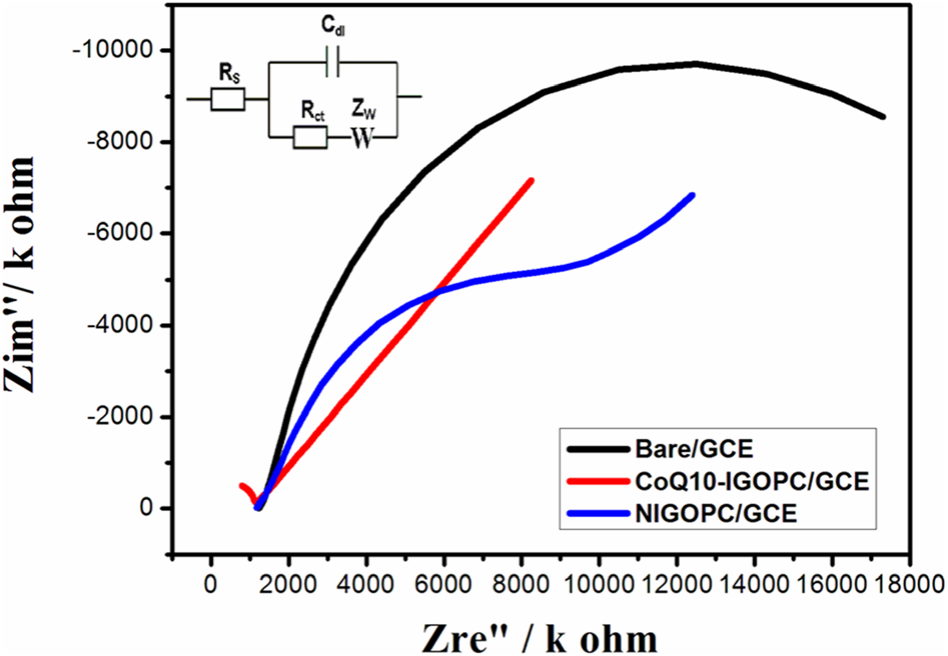
Electrochemical impedance spectrum of bare GCE, NIGOPC/GCE, and CoQ10-IGOPC/GCE.

The EIS provides crucial information on the impedance changes of the electrode surface due to its great sensitivity. EIS method was used in order to study the electrochemical response of electrode–electrolyte interface by the Nyquist plots of the bare, NIGOPC, and CoQ10-IGOPC modified GCE electrodes in 0.1 M KCl and 5 mM potassium hexaferrocynide (K_3_Fe(CN)_6_ and (K_4_Fe(CN)_6_) as electrolytic medium. In Nyquist plots the imaginary impedance (*Z*′′) was plotted against the actual impedance (*Z*′), which comprised a semicircle and a linear component. The electrode transfer restricted process was represented by the semicircle part at high frequencies, while the diffusion process was represented by the linear portion at low frequencies. The electron transport resistance at the electrode surface was equal to the diameter of the semicircle. It can be seen that the semi-circle of CoQ10-IGOPC/GCE is smallest in comparison with bare and NIGOPC modified electrode that further confirmed the conductive nature of CoQ10-IGOPC modified GCE electrode (Fig. 4). The obtained resistance values were 1218 ohms for bare 1154 ohms for NIGOPC and 0.001 ohms for CoQ10-IGOPC that clearly justified that the modified electrode had lowest resistivity as compared to the bare and NIGOPC/GCE (Fig. 4). Here, it is worth mentioning that imprinting on the surface of intercalated GO is producing more efficient electrode materials because they are efficient and swift in electron tranfer process.

### 3.4. Scan rate study of modified electrode

**Fig. 5:**
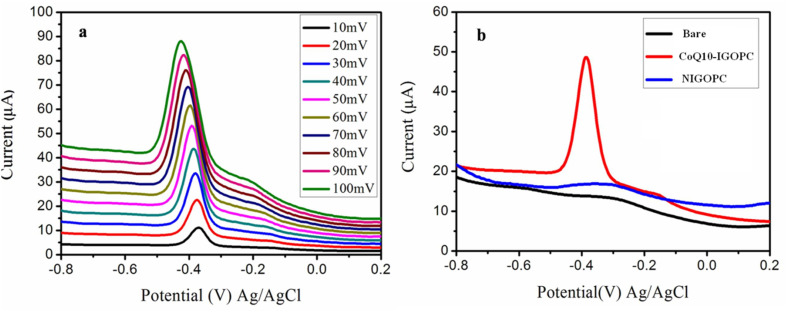
(a) Electrochemical response of CoQ10-IGOPC/GCE in the presence of 20.0 μM CoQ10 at different scan rates (from 10 to 100 mV s^−1^) in NaOH of pH 12. (b) Electrochemical response of bare GCE, NIGOPC/GCE, and CoQ10-IGOPC/GCE for 20.0 μM CoQ10.

In the presence of 20.0 μM CoQ10, the electron transfer behavior of the CoQ10-IGOPC/GCE was studied by measuring the current response of the DPV at different scan rates ranging from 10 to 100 mV in NaOH solution of pH 12. The reduction peak current of CoQ10 increased linearly with the increase in scan rate, the peak was moved towards negative potential, showing the conducting nature of the electrode due to graphene sheets enveloped in conducting copolymer of fluorene (Fig. 5a). Furthermore, peak currents were proportional to the square root of the scan rate, with an *R*^2^ of 0.991 suggesting that the electrode progress was mostly diffusion controlled. The presence of imprinted binding sites for CoQ10 on the surface of CoQ10-IGOPC/GCE allowed it to undergo electrochemical reduction of CoQ10 specifically.

### 3.5. Choice of electrode for the determination of CoQ10

The electrochemical redox reaction greatly depends on the ability of working electrode to offer current flow. The surface condition of electrode greatly influences its performance. The thin and porous surfaces allow enhanced and swift electron transfer kinetics and produce enhanced redox peaks.^30^ Preliminary studies were carried out to determine the general characteristics of bare/GCE, CoQ10-IGOPC/GCE, and NIGOPC/GCE, towards the electrochemical response of 20.0 μM CoQ10 in NaOH of pH 12 electrolyte solution (Fig. 5b). The bare GCE, and NIGOPC/GCE had not shown the response of CoQ10 in the potential range of −0.8 to +0.2, where as a significant reduction response of the CoQ10 was observed on the surface of CoQ10-IGOPC/GCE. It clearly indicated the better electron transfer kinetics of CoQ10-IGOPC/GCE due to expanded and porous surface of CoQ10-IGOPC and presence of conducting graphene backbone. The selective nature of CoQ10-IGOPC/GCE is due to the presence of imprinted binding sites for CoQ10 into its structure. Hence the CoQ10-IGOPC/GCE is best choice for the electrochemical detection of CoQ10 due to its specific imprinted cavities and larger active surface area of electrode.

### 3.6. Effect of electrolyte and pH solutions

**Fig. 6:**
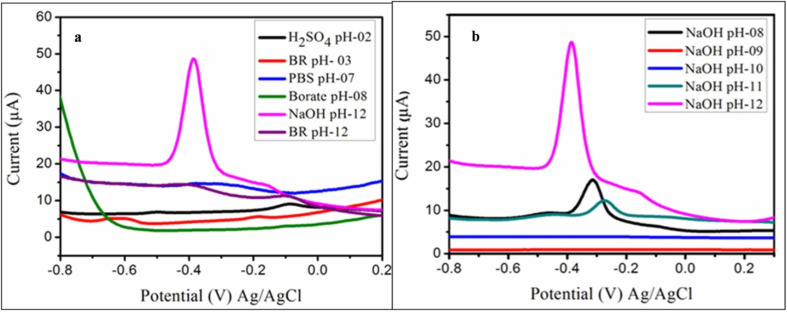
Electrolytic response of CoQ10-IGOPC/GCE in the presence of 20.0 μM CoQ10 (a) in different electrolyte solutions, and (b) at different pH of NaOH.

For the detection of CoQ10 the electrochemical response of CoQ10-IGOPC/GCE was significantly altered by changing the composition of electrolyte solution. Therefore, different supporting electrolytes *i.e.* sulphuric acid (H_2_SO_4_), sodium hydroxide (NaOH), phosphate buffer saline (PBS), borate buffer and Britton–Robinson buffers (BRB) were evaluated. The CoQ10-IGOPC/GCE showed no current response towards CoQ10 in all the above mentioned electrolytes except NaOH in between −0.8 and +0.2 V. A significant reduction response with sharp peak of CoQ10 was observed in case of NaOH electrolytic solution as shown in Fig. 6a. Therefore, the NaOH electrolytic system was chosen as optimal condition for the electrochemical detection of CoQ10.

The influence of pH of electrolyte solution was also optimized for the voltammetric detection of CoQ10 using CoQ10-IGOPC/GCE. The NaOH electrolytic solution of different pH (8.0–12.0) was studied to obtain the reduction response of 20.0 μM CoQ10. The results revealed that CoQ10-IGOPC/GCE did not show the response for CoQ10 at pH 8.0 and 9.0 whereas the little response was obtained at pH 10 and 11 but the intense reduction peak current response was observed at pH 12.0 as in Fig. 6b. Hence CoQ10 showed a strong current response on the proposed electrode at pH 12.0, it was chosen to be selective for further optimization studies. The CoQ10-IGOPC/GCE produced pronounced reduction peak of CoQ10 in NaOH because it is a strong base that leads to strong deprotonating of itaconic acid in poly(fluorene-*co*-itaconic acid) at the surface of GCE.^31^ The deprotonated CoQ10-IGOPC at the surface of GCE efficiently carries reduction of CoQ10 to ubiquinol-10 and produces sharp reduction peak.^32^ The phenomenon is more prominent at pH 12 of NaOH because of maximum deprotonation of itaconic acid functionalities.

### 3.7. Stability study

**Fig. 7:**
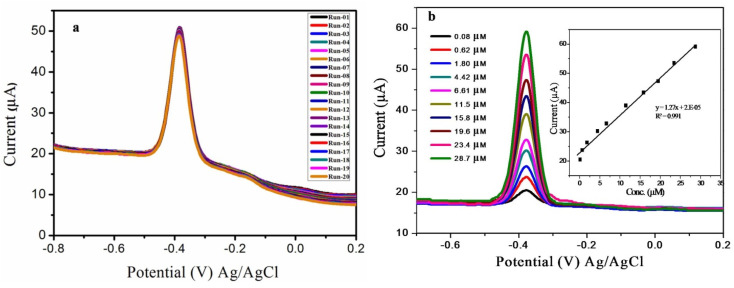
(a) Current response of CoQ10-IGOPC/GCE in the presence of 20.0 μM CoQ10 for twenty measurements, and (b) DPV response of CoQ10-IGOPC/GCE in the presence of different concentrations of CoQ10 (from 0.08 to 28.7 μM) in NaOH solution of pH-12 inset calibration graph for the quantitative detection of CoQ10.

The stability of the proposed electrode was evaluated by taking 20 cycles of DPV in NaOH (pH 12.0) electrolytic solution containing 20.0 μM CoQ10 (Fig. 7a). The obtained responses showed a great resemblance between the peak currents with (relative standard deviations) RSD < 0.66% indicating the good stability of sensor. The inter-day and intra-day stability of the proposed sensor was evaluated for ten days. The obtained recoveries of spiked samples were fairly good, and the RSD of the repeated quantification were not exceeded from 1% showing excellent repeatability of sensor. Over the course of two weeks, the reduction peak current of CoQ10 decreased as a result of repeated DPV tests. This is may be due to the deterioration of specific binding sites at the surface of CoQ10-IGOPC with passage of time *via* electrochemical process. Moreover, reproducibility of the fabricated sensor was evaluated by fabricating three identical electrodes. The current response of these three newly fabricates were taken by DPV which showed excellent reproducibility for the detection of CoQ10 with RSD of <5%.

### 3.8. Calibration plot of CoQ10-IGOPC/GCE V/S varied concentrations of CoQ10

The calibration curve was drawn using all the optimized conditions and parameters in NaOH solution of pH 12. For the calibration curve DPV responses were recorded at proposed CoQ10-IGOPC/GCE in the different concentration range of 0.08 μM to 28.7 μM of CoQ10. Fig. 7b showed that by increasing the concentration of CoQ10 the reduction peak current was also increased with a correlation value of *R*^2^ 0.991, a linear calibration curve was produced between reduction peak currents and molar concentrations of CoQ10. The LOD = 3(SD/*b*) and LOQ = 10(SD/*b*) formulae were used to calculate the limit of detection and quantification (LOD and LOQ). The ‘SD’ stands for the standard deviation of the intercept and the slope of the calibration curve is ‘*b*’ in these equations. The LOD and LOQ of the developed method were 0.029 μM and 0.0967 μM, respectively. The list of analytical figure of merits of the developed electrochemical method is given in Table 1. Moreover, analytical performance of the developed electrochemical method was compared with previously reported electrochemical and spectroscopic methods (Table 2). The manganese oxide modified screen printed electrode was reported to possess LOD of 64 × 10^−6^ M for CoQ10. The disposable electrode was able to detect CoQ10 and α-lipoic acid simultaneously but appeared to be less sensitive.^14^ E. V. Petrova *et al.* also reported a simple voltammetry method. However, the method could not reach the sensitivity offered by chromatographic methods.^32^ The compromised sensitivity of electrochemical methods for the determination of CoQ10 is their limitation. The chromatographic methods were also explored extensively to determine CoQ10. For instance, the reverse phase HPLC UV based method was proposed for determination of CoQ10 in hybrid nanoparticles. The method showed LOD of 23 × 10^−6^ M.^33^ More recently, a HPLC method based on electrochemical detection was reported for determination of CoQ10 in tissues, urine, plasma, *etc.* The hyphenation of HPLC with electrochemical detection resulted in extraordinary sensitive method with LOD of 2 × 10^−8^ M.^34^ In another study a HPLC method was reported for determination of CoQ10 from cheese. The method was found good in terms of selectivity with LOD of 2.78 × 10^−8^ M.^35^ However, methods based on HPLC need extensive sample preparation and are time consuming. The other methods for determination of CoQ10 include fluorimetric detection.^36^ Compared to the reported studies the current approach shows low detection limit with excellent accuracy. Hence the developed electrochemical approach could be best alternative over reported electrochemical, and laborious spectroscopic methods due its superior performance.

**Table 1:** List of analytical figures of merit of the developed electrochemical method for the quantitative detection of CoQ10

S. no.	List of analytical figures	Values
1	LOD	2.90 × 10^−8^ M
2	LOQ	9.67 × 10^−8^ M
3	Linear range	0.08–28.7 μM
4	*R* ^2^ (linearity)	0.991
5	Slope (*a*)	1.27
6	Intercept (*b*)	2 × 10^−5^

**7**	**Intraday precession**	
(a)	1.8 μM (*n* = 03)	0.34%
(b)	11.5 μM (*n* = 03)	0.30%
(c)	23.4 μM (*n* = 03)	0.33%

**8**	**Interday precession**	
(a)	1.8 μM (*n* = 09)	0.21%
(b)	11.5 μM (*n* = 09)	0.39%
(c)	23.4 μM (*n* = 09)	0.68%

**Table 2:** Comparative study of developed method with reported methods^a^

Technique	Material	Linear range	*R* ^2^	LOD	Matrix	RSD (%)	Ref.
HPLC	RP-column	0.57 × 10^−3^ to 28 × 10^−6^ M	0.999	23.1 × 10^−6^ M	CoQ10 co-encapsulated in lecithin/chitosan NPs nanoparticles	<2	19
HPLC/ED	RP-column	6 × 10^−8^ to 7 × 10^−6^ M	0.999	2 × 10^−8^ M	Tissues	<8	34
HPLC	RP-column	9.4 × 10^−7^ to 2.34 × 10^−6^ M	0.998	2.78 × 10^−8^ M	Cheese	<2	35
Fluorimetry	Magnetoliposomes	3 × 10^−8^ to 5 × 10^−7^ M		8 × 10^−9^ M	Vegetables, animal liver	<5	36
SWASV	MnO_2_/SPGEs	2.3 × 10^−6^ to 86.8 × 10^−6^ M	0.990	64.0 × 10^−6^ M	Pharmaceutical preparation	<5	12
DPV	GCE	10.0 × 10^−6^ to 1.00 × 10^−3^ M	0.999	3.33 × 10^−8^ M	Pharmaceutical preparation	<5	20
DPV	CoQ10-IGOPC/GCE	0.08 × 10^−6^ to 28.7 × 10^−6^ M	0.991	2.9 × 10^−8^ M	Pharmaceutical preparation, nuts and fruits	<5	Current study

a HPLC; high performance liquid chromatography, SWASV; square wave anodic stripping voltammetry, DPV; differential pulse voltammetry, RP; reverse phase, MnO_2_/SPGEs; manganese(iv) oxide-modified screen-printed graphene electrodes, GCE; glassy carbon electrode, CoQ10-IGOPC/GCE; CoQ10 imprinted graphene oxide base polymer-composite modified glassy carbon electrode, NPs; nanoparticles.

### 3.9. Selectivity of CoQ10-IGOPC/GCE towards the CoQ10

**Fig. 8:**
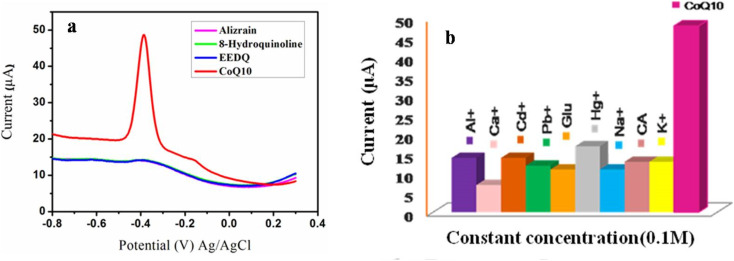
(a) DPV response of 20.0 μM CoQ10 at CoQ10-IGOPC/GCE in the presence of some structurally similar compounds, and (b) ionic interference for the detection of CoQ10 by CoQ10-IGOPC/GCE.

Specificity is the ability of method to response the target analyte in the presence of all the possible interfering species. In order to evaluate the specificity of CoQ10-IGOPC/GCE toward the CoQ10 the electrochemical response of three structurally resembling compounds including alizarin, 8-hydroquinoline (8-HQ), 2-ethoxy-1-ethoxycarbonyl-1,2-dihydroquinolin (EEDQ), and excipients of the pharmaceutical preparations such as glucose, citric acid, aluminum, calcium, cadmium, mercury, lead, sodium, and potassium were studied in same and 10 times higher molar concentrations, respectively (Fig. 8a and b). As given in the Fig. 8 minimum current response was observed in the presence of these species and a significant reduction peak current response was obtained by the addition of 20.0 μM CoQ10. The results showed that the presence of these excipients in the pharmaceutical preparations and structurally similar compounds had no interference effect for the detection of CoQ10 as given in Table 3 which clearly justifies that the fabricated sensor is highly specific towards CoQ10 due to the effective imprinting of CoQ10 in poly(fluorene-*co*-itaconic acid).

**Table 3:** Interference% of 10 times higher concentration of interfering species using CoQ10-IGOPC

S. no.	Interfering species	Conc. (μM)	Interference (%)
01	Glucose	200	23
02	Citric acid	200	25
03	Aluminum	200	28
04	Calcium	200	11
05	Cadmium	200	28
06	Lead	200	25
07	Mercury	200	32
08	Sodium	200	23
09	Potassium	200	26
10	CoQ10	20	—

Moreover, the selectivity of CoQ10-IGOPC/GCE was also confirmed by calculating imprinting factor (IF). IF is used to determine the selectivity of synthesized imprinted material on the basis of interaction between the functional monomer and template, higher the IF value the stronger is the interaction between the functional monomer and template and the lower the IF value the weaker is the interaction.^27,37^ IF of CoQ10-IGOPC/GCE was calculated by analyzing the homologues compounds of CoQ10 such as alizarin, 8-HQ and EEDQ (Table 4). The imprinting factor was calculated using equation given below. The calculated results revealed that CoQ10-IGOPC/GCE is 8.9, 8.5, and 8.2 times more selective for CoQ10 than alizarin, 8-HQ, and EEDQ, respectively due to the presence of specific binding sites that recognize the target molecule CoQ10.
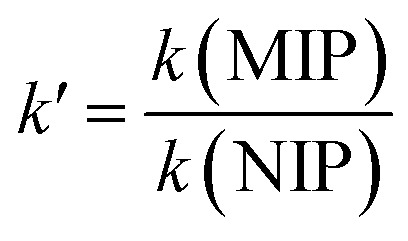
where *k*′ is the relative coefficient of selectivity and *k* is the coefficient of selectivity.

**Table 4:** Selectivity of CoQ10-IGOPC/GCE for CoQ10 in the presence of structurally similar compounds

S. No.	Compounds	MIP (μA)	NIP (μA)	CoQ10 competitor (μA)		
1	CoQ10	4.49	1.67	*k*_MIP_	*k*_NIP_	*k*′ = *k*_MIP_/*k*_NIP_
2	Alizarin	1.41	4.69	3.18	3.56	8.9
3	8-Hydroquinoline	1.42	4.47	3.16	3.74	8.5
4	EEDQ	1.41	4.32	3.18	3.87	8.2

### 3.10. Accuracy and precession

The proximity of the real values to the observed analytical findings is characterized as the method's accuracy. The method's accuracy was assessed by calculating the percentage relative error between observed mean values and actual values.^38^ The method's precision is measured in terms of percent RSD.^39^ The DPV response of CoQ10 tablet and capsules were obtained under optimal conditions to test the correctness of the established technique. The response of current in these matrices containing analyte was compared with the electrochemical response of standard CoQ10. Moreover, the precession of the method was evaluated by repeating the each measurement at least five times. The developed electrochemical method's accuracy and precision values were less than 5%, indicating that the method has excellent precision and accuracy for the quantitative detection of CoQ10 (Table 5). As a result, the proposed electrochemical technique may be used to detect CoQ10 quantitatively in pharmaceutical formulations.

**Table 5:** Application of the developed electrochemical method for the quantitative determination of CoQ10 in biological samples using CoQ10-IGOPC/GCE^a^

Sample	Claimed amount (mg)	Found (mg)	Recovery (%)	RSD (%)
CQG1	11.5	11.14	99.64	2.31
CQG2	11.5	11.12	99.92	3.84
CQG3	11.5	11.26	98.46	1.98
MCQ1	11.5	11.36	99.36	2.46
MCQ2	11.5	11.24	100.94	3.61
MCQ3	11.5	11.46	99.66	2.83
NCQ1	11.5	11.04	98.04	1.49
NCQ2	11.5	11.20	99.20	3.17
NCQ3	11.5	11.26	98.26	2.72

a CQG-CoQ10 gel capsule, MCQ-CoQ10 capsule and NCQ-Neo CoQ10 capsule.

## 4. Application of developed method

The concentration of CoQ10 was measured in the fruit, vegetables and nut samples to assess the practical applicability of the proposed electrochemical technique. The fruit (orange and strawberry), vegetables (spinach and tomato) and nut (peanuts and pistachios) samples were purchased from Jamshoro, Sindh, Pakistan's local market. The collected samples were prepared using reported method.^38^ To determine the % recoveries, all of the samples were spiked with three different known doses of CoQ10, that were found in the range of 98 to 102% RSD < 4% (*n* = 5) as given in the Table 6. The concentration of CoQ10 was detected as 12.2, 11.1, 8.1, 2.1, 6.6, and 1.2 μM in peanuts, pistachios, spinach, tomato, orange, and strawberry, respectively; however, all these values were within the standard range. The modified sensor was also applied for blood serum analysis of CoQ10. The blood samples of three healthy volunteers were taken and the serum was extracted by reported method.^38^ The electrochemical detection of CoQ10 in blood serum was carried out by using CoQ10-IGOPC/GCE. Table 7 shows the obtained results that are 0.7, 0.9 and 0.6 μM in S1, S2 and S3, respectively with % recovery range of 93.2 to 103.7% having RSD < 5%. The real samples analysis indicated that the suggested electrochemical sensor has exceptional capacity to detect CoQ10 quantitatively in real samples. Hence, the developed CoQ10-IGOPC/GCE sensor will be a promising option for selective CoQ10 detection with fast response and high accuracy.

**Table 6:** Determination of CoQ10 in fruit samples using CoQ10-IGOPC/GCE

Sample	Added (μM)	Found (μM)	Recovery (%)	RSD (%)	Sample	Added (μM)	Found (μM)	Recovery (%)	RSD (%)
Strawberry	0	1.27	—	1.97	Peanuts	0	12.17	—	2.34
1	5	6.24	99.45	3.65	1	5	17.12	99.73	3.21
2	10	11.29	100.14	2.84	2	10	22.2	100.03	2.51
3	15	16.33	100.34	2.93	3	15	27.5	102.2	1.96
Orange	0	6.60	—	2.65	Pistachios	0	11.12	—	1.53
1	5	11.7	102	3.97	1	5	16.14	100.4	4.56
2	10	16.58	99.85	4.46	2	10	21.5	103.8	3.79
3	15	21.72	100.8	3.24	3	15	26.6	103.2	2.83
Spinach	0	8.1	—	2.98	Tomato	0	2.1	—	2.65
1	5	12.9	96.71	3.17	1	5	7.2	102	3.12
2	10	18.21	101.1	3.66	2	10	12.3	100.3	3.49
3	15	23.4	102	3.94	3	15	17.22	100.8	3.54

**Table 7:** Determination of CoQ10 in serum samples using CoQ10-IGOPC/GCE. Quantitative detection of CoQ10 from serum samples by developed electrochemical method

Sample (serum)	Added (μM)	Found (μM)	Recovery (%)	RSD (%)
S1	0	0.7	—	1.92
1	5	5.4	94.74	2.65
2	10	10.3	96.26	4.84
3	15	16.1	102.55	3.93
S2	0	0.9	—	1.24
1	5	5.5	93.22	2.54
2	10	10.6	97.25	3.84
3	15	16.5	103.77	3.9
S3	0	0.6	—	1.21
1	5	5.3	94.64	2.54
2	10	10.2	96.23	4.33
3	15	16	102.56	3.15

## 5. Conclusion

This work reports cost effective and sensitive electrochemical sensing material for the detection of CoQ10. The fabricated CoQ10-IGOPC was designed to enhance the specificity and sensitivity of resulting electrochemical method. Therefore, CoQ10 imprinted poly(fluorene-*co*-itaconic acid) was fabricated into the layers of GO. The resulting CoQ10-IGOPC/GCE efficiently carried the reduction of CoQ10 at its surface in NaOH of pH 12. The developed electrochemical method was thoroughly evaluated for its application. The real samples of pharmaceuticals, vegetables, fruits, nuts and blood were analyzed to check the validity of developed method and it was found that developed CoQ10-IGOPC based electrochemical method can be efficiently employed for the detection of CoQ10 in complex matrices. Therefore, the developed method is recommended for the quantification of CoQ10 in pharmaceutical and food industry due to its specificity, sensitivity, cost effectiveness and robustness. Furthermore, the method is equally efficient for quantification of CoQ10 in biological samples and hence can be employed for clinical testing of CoQ10.

## Conflicts of interest

All the authors of this manuscript confirm that there is no conflict of interest.

## Supplementary Material

RA-012-D2RA05401A-s001
