# Supplementary material for: Fluorene intercalated graphene oxide based CoQ10 imprinted polymer composite as a selective platform for electrochemical sensing of CoQ10
Source: RSC Adv. 2022 Nov 4;12(49):31639–49. doi: 10.1039/d2ra05401a (PMC9634718; doi:10.1039/d2ra05401a)
Supplement: RA-012-D2RA05401A-s001 [file RA-012-D2RA05401A-s001.pdf]

Electronic Supplementary Material (ESI) for RSC Advances.
This journal is © The Royal Society of Chemistry 2022

Flourene intercalated graphene oxide based CoQ10 imprinted polymer composite as selective platform for electrochemical sensing of CoQ10

Anam Naz Soomro[1], Huma Shaikh[1,*], Mohammad Imran Malik[2], Jamil A. Buledi[1], Sehrish Qazi[1], Amber Solangi[1]

[1]National Center of Excellence in Analytical Chemistry, University of Sindh, Jamshoro-76080, Sindh, Pakistan

[2]H.E.J. Research Institute of Chemistry, International Centre for Chemical and Biological Sciences (ICCBS), University of Karachi, Karachi-75270, Sindh, Pakistan

2.1S. Extraction of CoQ10 from real samples:

Sample preparation was carried out by taking 2g Peanut, Pistachio, tomato and Spinach (with 5ml saline solution) and 2g Strawberry and Orange (without saline solution). All samples were homogenized in an extraction tube. After homogenization, 8 ml of ethanol was added to the solutions. Following that, 20 ml n-hexane was poured to a tube and agitated violently for 10 minutes, to separate the layers, the tube was immediately centrifuged. The bottom layer was re-extracted two to three times using (1:4) ratio of ethanol and hexane. After that hexane layer was preserved. The mixed n-hexane layer was evaporated by putting the sample in fuming hood for a night, and the residue was then dissolved in 5ml acetonitrile [1,2]. The quantity of CoQ10 in soft-shelled capsules was measured by dissolving entrapped oil in organic solvent (0.05 percent BHT solution in n-hexane and THF in 95:5 ratios). The samples were sonicated for 10 minutes, shaken for 30 minutes, and then centrifuged for 10 minutes at 5000 rpm. About 5 ml of the clear supernatant was evaporated to dryness under a stream of nitrogen and were reconstituted in 1 ml of 0.01 percent BHT solution in acetonitrile for complete CoQ10 evaluation[3]. In a centrifuge tube containing 2.0 ml of H_3PO_4, one tablet (powdered) was added and subjected to vortex for 10 minutes. The samples were poured and sonicated in organic solvent (8.0 ml of 0.05 percent

solution of ascorbic acid in n-hexane). Samples were centrifuged at 5000 rpm for 10 mints. The clear supernatant fraction of 5 ml was evaporated to dryness under a stream of nitrogen and reconstituted in 1 ml of 0.01 percent BHT solution in acetonitrile[4].

While for the extraction of blood serum the whole blood was collected in commercially available tubes (red-topped tubes) and was allowed to clot by keeping it undisturbed at ambient temperature for 15–30 minutes. The clot was centrifuged at 1000–2000 rpm for 10 minutes that results the serum in the form of supernatant. After centrifugation, the serum was quickly transferred into a clean polypropylene tube. During handling, the samples were kept between 2–8°C[5].

References

1. P. Mattila, M. Lehtonen and J. Kumpulainen, Journal of Agricultural and Food Chemistry, 2000, 48, 1229-1233.
2. P. Mattila and J. Kumpulainen, *Journal of Food Composition and Analysis*, 2001, **14**, 409-417.
3. Ž. T. Rakuša, E. Srečnik and R. Roškar, *Acta Chimica Slovenica*, 2017, **64**, 523-529.
4. Ž. Temova Rakuša, E. Srečnik and R. Roškar, *Acta Chimica Slovenica; Vol 64, No 3 (2017)*, 2017, DOI: 10.17344/acsi.2016.2856.
5. P. Thavasu, S. Longhurst, S. Joel, M. Slevin and F. Balkwill, *Journal of immunological methods*, 1992, **153**, 115-124.
